# The varying social dynamics in orally transmitted and notated vs. improvised musical performance

**DOI:** 10.3389/fpsyg.2023.1106092

**Published:** 2023-04-20

**Authors:** Tamar Hadar, Tal-Chen Rabinowitch

**Affiliations:** ^1^The School of Creative Arts Therapies, University of Haifa, Haifa, Israel; ^2^Department of Education, Western Galilee College, Acre, Israel

**Keywords:** tight-loose theory, musical genres, improvisation, social interaction, musical performance

## Abstract

Musical performance can be viewed as an intricate form of social behavior. Accordingly, the rich diversity of existing musical styles and traditions may reflect distinct modes of social interaction. To gain a better understanding of the relations between musical style and social dynamics, we have formulated a framework for dissecting different genres of musical performance according to key social criteria. In particular, we contemplate on the continuum ranging from strictly orally transmitted and notated to fully improvised music, and its relation to general compliance with social norms and structure, borrowing key concepts from tight-loose theory, a powerful paradigm for studying societal behaviors and tendencies. We apply this approach to analyze four distinct prominent musical genres, providing a detailed mapping between musical style and social dynamics. This work highlights important factors that link between musical performance and social interaction, and will enable future experimental unraveling of social aspects of musical performance as expressed by different musical styles and practices.

## Introduction

Different styles of musical performance demand varying levels of accuracy and planning (Davies, 2001; Thom, 2020). Thus, notated and orally transmitted music, compared to improvised music, for example, requires a high level of alignment between players, careful compliance with written notes and instructions, and a systematic level of playing that adheres to the composer’s intentions (Thom, 2020). Given that musical performance can be considered as a complex form of social interaction (D'Ausilio et al., 2012; Keller et al., 2014; Robledo et al., 2021), it stands to reason that performance styles should associate with congruent forms of social dynamics. In particular, we would like to propose that whether music performance is based more on notation and oral tradition or more on improvisation, may correspond to the degree of adherence to more general social norms displayed by the social dynamics incorporated within the music-making.

To examine and develop this notion we analyze several established traditions of music performance with respect to distinct criteria of social dynamics along the axis ranging from tight to loose social compliance with norms, a framework originally developed for studying societal behavior (Pelto, 1968; Gelfand et al., 2011). This analysis brings us closer to understanding joint music-making as a social phenomenon and its relations to particular forms of social dynamics.

We wish to emphasize that our main aim was to capture varied social dynamics that underlie different types of musical performances and to strengthen our theoretical understanding of this phenomenon. To this end we chose four different musical traditions that serve to exemplify the social components of joint music performances. Therefore, rather than providing a comprehensive musicological or ethnomusicological account of the musical genres explored, we focus on particular tendencies of music engagement that they represent with an emphasis on their underlying social dynamics. We thus intend to treat each musical genre as an ideal prototype of a particular mode of musical interaction. These include: (1) Western classical; (2) Hindu music; (3) Jazz; (4) Free jazz improvisation. These four musical genres span a broad representational array of performance conditions which allowed us to explore an extensive continuum of orally transmitted versus improvised traditions, along the lines of Kay (2016):

“If freely improvised music and creative jazz lie at one end of a spectrum, then the other end would inevitably be secured by Western classical music, where there is variation only in the interpretation of a defined composition. I would place North Indian raga music in the middle of the spectrum: it balances in harmony the best attributes of classicism, retaining the continuity of past and tradition while respecting artists’ intuitive and improvised interpretation of the present” (p. 10).

We therefore wish to further explore the continuum proposed by Kay (2016) and to add an analysis of the social structures underlying it.

## Mapping between performance attributes and social structure

### Western classical music

Western classical music encompasses an era stretched over 1,000 years of diverse styles and traditions originating in the Latin plainchants of the Roman Catholic Church. The chants did not include any musical instruments which were forbidden by the church, and were exclusively monophonic (Taruskin, 2010; Levy et al., 2022). Though writings and drawings from that time imply the existence of secular instrumental music, which is believed to have incorporated polyphony, no remaining notated scores have been found to confirm this hypothesis (Taruskin, 2010). Modern writings about the performance of plainchants (e.g., Berry, 1979; Brunner, 1982) highlight the essentiality of maintaining an authentic performance, one that is consulted with experts of medieval music and is not bound to mainstream assumptions regarding this tradition (Brunner, 1982). Such historic approaches, which remain alert for novel directives to uncover authentic traditional musicianship, imply a broader social influence of a millennium-practiced musical tradition. In other words, it illuminates additional possible structures presumably remaining untouched, other than the musical ones (e.g., the social structures between the players and/or the players and audience, see more in Small, 1998).

The first secular music documented is known to be the Troubadour songs, sung by poet-musician knights. Located in today’s modern France, Aquitine, this elite tradition emerged, introducing love and knigtly songs (Taruskin, 2010). Though not bound to religious controls, according to Taruskin (2010), this musical style reflected strong social norms of feudalism and hierarchy, with its harmonious and ideal poems.

Although presumably existing well before, polyphony became a central practice in European music only during the 12^th^ century (Taruskin, 2010; Frobenius et al., 2022). This period also marks the establishment of meter and notation as essential components of composition and performance (Taruskin, 2010). The next musical form created during the 13^th^-14^th^ century was the French Motet. The Motet, a vocal composition that emerged as a Latin religious practice but cultivated to include secular branches, enabled composers to further elaborate harmonic techniques, and complex writing of several melodies including text (Taruskin, 2010).

From the secular motets of Dufay Guillaume de Machaunt, a renowned composer of the 13^th^ century, Western classical music evolved into the early Renaissance period, giving rise to the famous pieces of Du Fay, which included masses, magnificents, motets and hymns, which imported English harmonic techniques and styles. A century later, the form of the Mass reached a peak, represented by one of its famous composers, Josquin des Pres (Sanders et al., 2022). In that time the Ordinary of the Mass was established including the Kyrie, Gloria, Credo, Sanctus, and *Agnus Dei*. Later Renaissance gave rise to the Madrigal form and mostly branched into either vocal work or compositions of instrumental dances. The Renaissance era fostered several performance practices. e.g., *cantare super librum* (Bent, 1983), that at first historical sight might be understood as types of improvisation. However, a closer examination of this type of performance revealed that what was casually considered by some researchers as a spontaneous and free expression (Ferand, 1957), was in fact “music fully or sufficiently conceived but nevertheless unwritten” (Bent, 1983, p. 378). Bent (1983) therefore argued for a more subtle definition for inventing musical lines on the spot, one which reflects the extremely sophisticated and strict set of rules underlying such musical practice. Bent’s approach emphasizes the strict nature not only of orally transmitted music, but also of the music that had greater degrees of freedom, i.e., *cantare super librum.* Such historic accounts support our central argument about the strict musical and social characters implicit to Western classical music. Conversely, Broude (2020) linked the shifting of *cantare super librum* practice from being solely improvised to exclusively notated to the rise of music-printing industry, the increase in amateur musicians who could not improvise counterpoint lines sufficiently and mostly related to our piece: to the materialization of the construct of “art works” (Broude, 2020).

1,600 to 1750 marks the Baroque era, represented iconically by the compositions of Johan Sebastian Bach and George Friedrich Handel. During the Baroque period the seeds of modern harmony and orchestration were planted, along with the idea of the modern orchestra and modern opera (Taruskin, 2010). Largely influenced by Italian music, the idea of solo voice for instruments was born. This is exemplified in Bach’s Cantatas. Handel’s famous “Messiah” oratorio represents a peak of compositional advancement as well as religious topics performed in secular halls.

This accentuates the expansion of music to additional realms of society. However, at the same time demonstrates the strong religious ties it continues to maintain to this day. The excitement and interest in different instrumental timbres and arrangements, e.g., the trio sonata, influenced composers’ attention and appreciation of the violin, which was considered the highest rank of all instruments (Sanders et al., 2022). This is highly notable in Vivaldi’s various violin concertos and sonatas. Different musical forms emerged, giving rise to great versatility and styles during the 17th–18th centuries, such as the Concerto and Fugue for example. The latter not only implies the centrality of instrumental music but also facilitated the emergence of novel compositional structures and techniques, e.g., deploying repetition of themes, exposition, change of key and more. However, the techniques mentioned flourished into their full potential during the Classical era, where structural clarity, symmetry and stability gained centrality over textural intricacy.

The Classical era was stretched between three different styles: the first, “Reform opera” (as in Gluck’s famous Orfeo ed Euridice), focused on the opera’s drama rather than on the singers’ solos. The second style evolving at the time related to the centrality of keyboard work. Moving from the harpsichord to the clavichord emphasized the emergence of great pieces such as Carl Philip Emanuel Bach’s keyboard sonatas. As mentioned above, this was also influenced by the significance of the Sonata form at the time (Mangsen et al., 2022). Along the centrality of piano-playing, various improvisational practices flourished in the Classical era, e.g., the Cadenza, the Fantasia and the prelude (Goertzen, 1996). According to Goertzen, while the Fantasia was a well-established practice, often reviewed and mentioned in concerts’ programs, the prelude remained somewhat in the margins of Western music. Goertzen claimed that one of the social-cultural movements that influenced the diminishing of such improvisational practice pertains to the rise of printed music and printed programs, leaving the spontaneously crafted parts of classical performance sealed in the past. The third musical style peaking at the time was the symphony (Larue et al., 2022). Originating in the Greek work “sounding together,” this musical style symbolized a new mode of relatedness and social balance in music (Larue et al., 2022). Along with being part of the Catholic service, in its early format, symphonies were present at different venues and concert halls: from official ceremonies to private concerts held in palaces and residencies of the local aristocrats (Larue et al., 2022). Represented iconically with Joseph Haydn, Wolfgang Amadé Mozart and Ludwig van Beethoven’s symphonies, this form has also bridged Western music into the Romantic era.

With Beethoven’s later work, along with composers such as Hector Berlioz, Franz Schubert, Frederick Chopin, Clara and Robert Schuman and many others, Western classical music entered the Romantic period. Key features of this time included greater emphasis on emotional expression, as opposed to the previous period’s focus on clarity and structure (Taruskin, 2010). At the same time, orchestras became bigger, and the harmonic structures and arrangements became more intricate and diverse. On the social level, Romanticism emphasized the “I,” calling for expressiveness and individualism, however, this “I” very quickly grew into the “big I” or, “We,” identifying a national component within the musical culture (Taruskin, 2010). The late 19^th^ century gave rise to novel compositional exploration such as the symphonic Lieder, as indicated in Gustav Mahler’s symphonies.

The early 20^th^ century is characterized by the emancipation of basic harmonic structures such as scales and form, as represented by Claude Debussy’s contributions on the one hand, and Igor Stravinsky’s compositions on the other. Later periods of the 20^th^ century incorporated many complex post-war and cold war influences (Taruskin, 2010; we will not be covering those in this article). The migration of many composers (e.g., Schoenberg, Stravinsky, Bartók, Hindemith, Krenek, Korngold, Milhaud and more) to America as a consequence of Hitler’s regime, influenced the meeting between European and American styles, which had a profound effect on the history of Western music (Taruskin, 2010).

Though it would have been of great significance to explore the delicate changes that have evolved in form and style throughout the Western classical eras, we focus on more fundamental aspects witnessed along the stretch of a millennium of Western classical music, i.e., on the constant presence of some kind of hierarchy (e.g., between players, instruments, styles), clear compositional forms, and a literate notation from as early as the 9th century. All the styles and iterations mentioned above have evolved from the establishment of defined musical roles between players (even if these roles are exchanged – this would be scripted by the composer), which we believe have created a distinct social matrix between the players.

At the same time, in Western culture, improvisation is popularly considered as branching out from strictly notated music. However, up until 150 years ago, the basic skills of any musician equally included performance, improvisation and composition (Moore, 1992). Indeed, improvisation was a fundamental part of Western Classical music evident over different eras: Renaissance, Baroque, Classical and early Romantic music. Moore (1992) raised the question of how improvisation suddenly vanished during the 19^th^ century and pointed at the lack of research on this striking phenomenon. As mentioned, one reason was that musicians became more specialized in specific roles (i.e., composer versus instrumentalist; Moore, 1992; Bailey, 1993; Goehr, 1994). However, perhaps the crucial driving force for notated music becoming predominant in Western society from the 19th century onwards was its embracement by the upper class, while improvisational music came to be regarded as less prestigious and was associated with the lower social classes (Moore, 1992; Fischlin and Porter, 2020). Thus, notated music became a symbol of social status, superseding other previous cultural functions of music making, such as creating communities and allowing a free, improvisatory relationship between performer and audience. As a consequence, musicians were increasingly encouraged to focus on composing music for the elite. Even musicians belonging to the margins of society at that time, now found an opportunity for acceptance in higher social circles, by emphasizing their compositions (Moore, 1992). Concomitantly, improvisation was pushed out of the mainstream of musicological research (Moore, 1992; Monson, 1996; Fischlin and Porter, 2020).

Perhaps the most crucial motivation for the transition from improvisation to notation in Western music was the effectiveness of notated music in preserving strict social hierarchies and values (Moore, 1992; Goehr, 1994; Small, 1998). Thus, it seems that the vanishing of musical improvisation in Western Classical music around the 19^th^ century was closely tied with contemporary social structures, thus is indicative of a strong link between musical style and its social underpinnings.

### Hindu music: Hindustani and Carnatic traditions

Prior to separating into its two central streams of Carnatic music and Hindustani music around the 12^th^ and 13^th^ centuries, Hindu music can be traced 2,500 years back, in the chant and utterances of the Vedic period: devotional hymns sang to the gods, also called *Samaveda* (Gowrishankar, 2022). The Hindustani stream is traditionally divided into four periods: Delhi sultanate, Mughal period, the Colonial era and Modern Hindustani. All four eras present rich musical developments with key musicians greatly influencing the establishment of diverse styles and forms. The Carnatic stream is acknowledged with four main periods as well: Vijayanagar period, Pre-Trinity period, Trinity period and Post-Trinity period. The Trinity period is considered the golden age of Carnatic music, referring to three great composers who substantially innovated the ragas and talas, establishing new forms of Carnatic music (Vijayakrishnan, 2007; Gowrishankar, 2022).

Though we will not be able to encompass the vast musical traditions evolving at different times, it is of great interest for us to realize that throughout almost a millennium the Hindu music of both streams preserved its connections with its ancient origins while innovating with ragas and other forms (Nettl, 2014; Gowrishankar, 2022). The magnitude and domination of the traditional aspects in Hindu music, we believe is fundamental to the social context created by Classical Indian performance. As specified by Nooshin and Widdess (2005), it does not only influence the players’ adherence to their musical role (e.g., leader or accompanist of the raga improvisation), but also creates strong expectations among the audience.

While the Northern tradition, i.e., Hindustani classical music stemmed from several ancient influences such as the ancient Persian tradition *Musiqi-e assil*, the Southern branch. i.e., Carnatic classical music, evolved under the influence of the reformative *Bhakti* movement (Vijayakrishnan, 2007). Unlike Hindustani music, Carnatic music emphasizes vocal music, whereas instrumental music is secondary and inspired by vocal characteristics (Vijayakrishnan, 2007). In addition, this branch did not incorporate any exclusive instrumental forms (Gowrishankar, 2022). Remaining unaffected by Islamic conquest of the Northern part of India, as well as departing from any Persian influences, the Carnatic stream remained truthful to ancient Hindu traditions. On the other hand, the Hindustani stream was conceived as a synthesis between traditions, the religious and the secular (Nettl, 2014). The three vocal streams identified with Hindustani music are *Dhrupad*, *Khayal*, and *Thumri.* Under the rule of Delhi Sultanate, important musicians such as Amir Khusrau evolved, who is identified with establishing and formalizing some of the basic tenets of Hindustani music. Though differing in many social and historical factors, the two branches do share some similarities in terms of forms and performance styles, i.e., the *Raga* (scale) and *Tala* (cyclic rhythmic pattern), though they developed differently in each stream – the Hindustani or the Carnatic (Nettl, 2014). An additional aspect shared by both Hindustani and Carnatic streams relates to the pre-determined roles of the musicians in the music ensemble (Nettl, 2014): the melodic soloist (present in both streams); and the accompaniments: string instruments (Sitar or violin); and a set of drums (*Tabla*). In all streams of Indian music, the soloist is considered the group’s leader and is almost exclusively engaged in improvising. The other players provide a rhythmic and drone basis, and at times participate in structured moments of improvisational dialogues with the soloist (Nettl, 2014). While in the Carnatic stream the voice gained great centrality, in the Hindustani music the instruments were more salient in the musical performance (Vijayakrishnan, 2007). Pertinent to our discussion of possible social structures embedded in varied musical traditions, are the strict roles of soloist versus accompanist, which remained untouched throughout almost three millennia.

When discussing the evolution of Hindu music, one should take into account the subtlety in which issues of social class are entwined within the performance and musical relationships of the musical tradition (Clayton and Leante, 2015; Alaghband-Zadeh, 2017). In Hindustani music, according to Clayton and Leante (2015), this social aspect can manifest either in pre-determined roles specific to the performance (e.g., soloist; accompanist; or listener), or in matters relating to the underlying relation between the players (e.g., teacher-student; family members). In this respect, the authors highlight several historical changes occurring throughout the 19^th^ century, one of them illustrated by many musicians of *Sāraṅgī*-playing families becoming solo singers and henceforth abandoning their accompanying instrument due to its association with low-status. Alaghband-Zadeh (2017) shows how ancient and active practices of “active expert listeners” in North Indian performances, preserve aspects of class, roles and aristocracy among the relationship between the players as well as between musicians and their audiences.

In her attempt to define the nature of Hindustani music, McNeil (2017) argues that the Hindustani musical tradition was interpreted inaccurately by western researchers, who used western terminologies and ontologies of improvisation and notation, thus imposing a binary system of thought that does not apply to Hindustani music. McNeil highlighted the difficulty in translating the term improvisation, which represents different realities and varied aspects of musical creativity in each culture. The segregated approach, typical to Western theory of music, fails to include central parts of Hindustani, as well as Persian and Arabic music, according to McNeil (2017). In his ethnographic exploration of his personal journey into Indian music, Kay (2016) compares performing a *Raga* to painting a portrait of a well-known and beloved personality that was not completely pre-planned. In other words, Kay captures in his description the traditional as well as the creative aspects of performing classical Indian music.

Nooshin and Widdess (2005) explained that both Iranian and ancient Hindu music did not theorize the term improvisation, but musicians rather dedicated some parts of the musical performance as responsibilities of the performers. The authors specified however that nowadays, the two main Classical Indian streams (i.e., Hindustani and Carnatic music) differ in that sense. While Hindustani musicians use terms such as *Bandis* and *Gat* to refer to pre-composed and relatively unchanged parts, and *Upaj* and *Khyal* as indicating innovative parts of the players, modern Carnatic music seems to allow a rougher distinction between pre-composed music, *Kalpita* (or sometimes known as *Kriti*), and music that is created on the spot, *Kalpana*.

Several authors have highlighted the emotional component of Hindustani classical music (Nooshin, 2003; Nooshin and Widdess, 2005; Pudaruth, 2016). According to Pudaruth, Hindustani music is “always making reference to and depicting some emotions outside itself… a music comprising various ‘channels’, through which a wide range of human emotions can be expressed” (p. 6). Creativity and emotional expression, she concludes, are essential for engaging in the Hindustani musical tradition. Pudaruth further explains that in Hindustani aesthetics, the artistic experience entails reaching a unique emotional state called *Rasa*. The *Rasa* is differentiated from a more daily experience of emotions in that it allows the beholder to experience the emotion in full, thus inducing a euphoric or ecstatic feeling of bliss. In those moments, the beholder becomes unified with the art form: there is no separation between knower and known, and there is no egocentric consciousness (Pudaruth, 2016). Thus, Hindustani music focuses on reaching a contemplative, transcendental and even spiritual state of mind.

Instead of the dualistic terms of improvisation and notation (the former does not even exist as a word in any of the languages used in India), McNeil (2017) framed her discussion about Hindi music performance, using the terminology of *Vistaar* and *Badhat,* suggesting feelings of expansion or growth, and *Upaj* and *Andaaz*, emphasizing creativity and novelty. Hindustani music, McNeil explains, incorporates only mild references to fixed structures, or what she conceptualizes as “seed ideas.” These seeds are manifested in three possible layers, defined by the raga itself, the song’s structure and the rhythmic patterns (*Tala*). Using culinary metaphors, McNeil explained that the challenge of the Hindustani musician is to realize the methods and strategies appropriate to the musical context that will allow them to complete the preparation of only “half-baked” musical materials. In other words, the so-called fixed elements of Hindustani music are only preliminary entities, which are subjected to varied interpretation and manifold possibilities to evolve. McNeil’s conceptualization of seed ideas not only serves the author in establishing strong arguments for understanding Hindustani music within an appropriate language and cultural context, but also helps distinguish this type of musical creativity from Western improvisation. As specified by McNeil, the *seeds* are present in the performance from the very start and are raised to their mature, developed potential, through the creative process unraveled by the musician, a process of expansion and growth. On the contrary, in Western music, musical elements such as the harmonic and melodic structure do not function as encapsulated primary forms, only waiting for the musician to bring them into life (hence the seeds) but reveal themselves as fixed musical structures that the musicians can play around with, stretch, expand, rebel and work against, to a certain extent (Berliner, 1994; Monson, 1996; Hadar and Amir, 2021).

### Jazz

Jazz was born in the U.S. in the early 1920’s, featuring a musical approach that highlights improvisation and creative processes, and specific artistic styles that include structural, rhythmic, melodic, harmonic and timbrical elements influenced partially by African American religious music (Lewis, 2002). It is important to note that not all jazz styles have the same emphasis on improvisation. The basic ingredients of a jazz tune (known as a standard) include: (1) a melody (usually referred to as head), and (2) a harmonic progression, usually referred to as changes (Berliner, 1994). The rich repertoire of jazz standards incorporated a diversity of musical influences, such as spirituals, marches, rags and popular songs (Berliner, 1994).

The common approach to a standard is playing the head in the opening and closing of a performance and playing solos in between, usually rotating between all members of the band. This, however, is only the basic way jazz players use the standard for their improvisatory exploration. The basic structure itself is often altered and personalized by the players, and even by the same player, after performing the same piece for many years (Berliner, 1994). For example, players often change the tonic (i.e., choose a different key to elicit new timbres), or occasionally alter the melodic feature to add more tension or to create different instrumentations. In addition, players make subtler changes to the basic structure, by using repressed or exaggerated vibrato; emphasizing slurring or tonging; adding accents in different places and finding novel sounds and timbres with their own instruments. Thus, jazz combines structure with improvisational content. The improvisational *solo* represents the main platform for freedom and exploration, and the structural choices hold a level of flexibility and a means for the musician to personalize their performance (Berliner, 1994).

In her expansive investigation of the music of African American culture, Monson (2007) established important links between the musical genre of jazz and the consolidation of the cry of Black people for equity and recognition. Monson poses critical questions such as:

“What effects, direct and indirect, did the struggle for racial equality have on aesthetics, the sense of mission musicians brought to their art, the diversity of music played and composed, and the symbolic meanings attached to the art form? What role did world affairs, especially African independence and anticolonialism, play in how African Americans came to envision their political and cultural liberation? In what ways did the ideas of aesthetic modernism mediate between music and politics?” (p. 4).

Thus, jazz has played an important social role of bringing forward the voice of African American people to the forefront of the musical scene (Bailey, 1993; Berliner, 1994; Monson, 1996, 2007). However, it seems that another form of improvisation, free jazz improvisation, was necessary for breaking through musical norms and past traditions.

### Free jazz improvisation

In free jazz improvisation^1^ performers do not establish any pre-determined frames or idioms to guide their joint playing. Free jazz improvisation evolved during the late 1950’s, as a post-war tradition (Pras, 2015) that rejected former jazz conventions, such as bebop, New-Orleans jazz and swing (Lewis, 2002; Pras, 2015). Those years set a historic landmark in the evolution of improvisation, and a point of intersection of culture, history, and music (Lewis, 2002). The tension between two social sectors gave rise to the novel trend: one representing African American culture, where jazz had originated; and the other subscribing to Western European movements, attempting to elide the fundamentality of Black culture to the formation of free jazz improvisation (Lewis, 2002). Within this heated atmosphere, the new musical genre was conceived as a strong rebellious musical medium (Lewis, 2002; Pras et al., 2017).

These significant years had a worldwide influence on the state of jazz and improvisation, leading to free jazz improvisation being incorporated and integrated into different musical traditions (Fischlin and Porter, 2020). Fischlin and Porter (2020) provide an international and multi-cultural view of how societies dealing (present and past) with colonialism, war and trauma use improvisation to build their independent voices and sense of freedom in a reality of terror and domination. Emphasizing the association between improvised musical genres and marginal socio-economic sectors of society, the authors capture the powerful strategies improvisation offers minorities to resist and fight oppression and hierarchal social structures.

In their collection of essays (Fischlin and Porter, 2020), the authors describe varied communities around the world, where improvisation was used as a strategic tool to provide individuals with an opportunity to reflect on the social structures perpetuating their ongoing suffering. Through the artistic practice of improvisation, they explain, people might find novel ways to express themselves and broaden their horizons, within restrictive and difficult environments, or in the authors’ words, improvisation elevates “the responsibility to act creatively and in concert with others to reclaim a public common under attack” (p. 11).

Zim Ngqawana’s Exhibition of Vandalism (Vos, 2020) for example, invited the audience to experience the consequences of social-political realities in South Africa, through engaging in a healing ritual of musical improvisation, a musical ritual that concurrently emphasizes destruction, reconciliation and growth. In this improvised response to vandalism, the players played both intact and vandalized instruments together, thus representing the distorted and convoluted social situation faced by the musicians. It should be noted that while free jazz improvisation seems to emphasize the individual and, at times marginal voices of society, and to promote modernism and breaking of rules and norms, other modes of improvisation also exist, such as from East Asian origins, which seem to highlight, instead, the musician’s connections to their cultural traditions. For example, when a musician performing classical Indian music is adhering to the sacred structure of the *raga* their playing (Pudaruth, 2016).

We commenced our paper by describing the complex histories of four musical genres (i.e., Western classical; Hindu music; Jazz; and Free jazz improvisation), while highlighting salient social intricacies pertaining to each genre. In the following part we will further deepen our examination of the social layers embedded in each musical genre. We will first set the stage for our central discussion regarding the social aspects of musical interaction (i.e., ‘A social lens to music’). We shall then focus our discussion on the social components as reflected in the genre’s placement along a continuum ranging from orally transmitted and prearranged music, to improvisational traditions. Next, we will introduce the Tight-Loose (i.e., T–L) framework, which we will deploy to examine the possible social meanings underlying various musical styles. In this respect we will specify four societal parameters, which capture the music’s behavior along the T–L continuum (i.e., structural sparseness; flexible social roles; cultural nonconformity; and creative freedom). Finally, we will summarize our findings in a Table 1 and discuss different possible consequences of the genres’ level of T–L on the social dynamics afforded between musicians (e.g., common group membership vs. tolerant group membership).

**Table 1:** Portrayal of each musical style according to 4 categories of social tightness/looseness and the average score reflecting overall looseness.

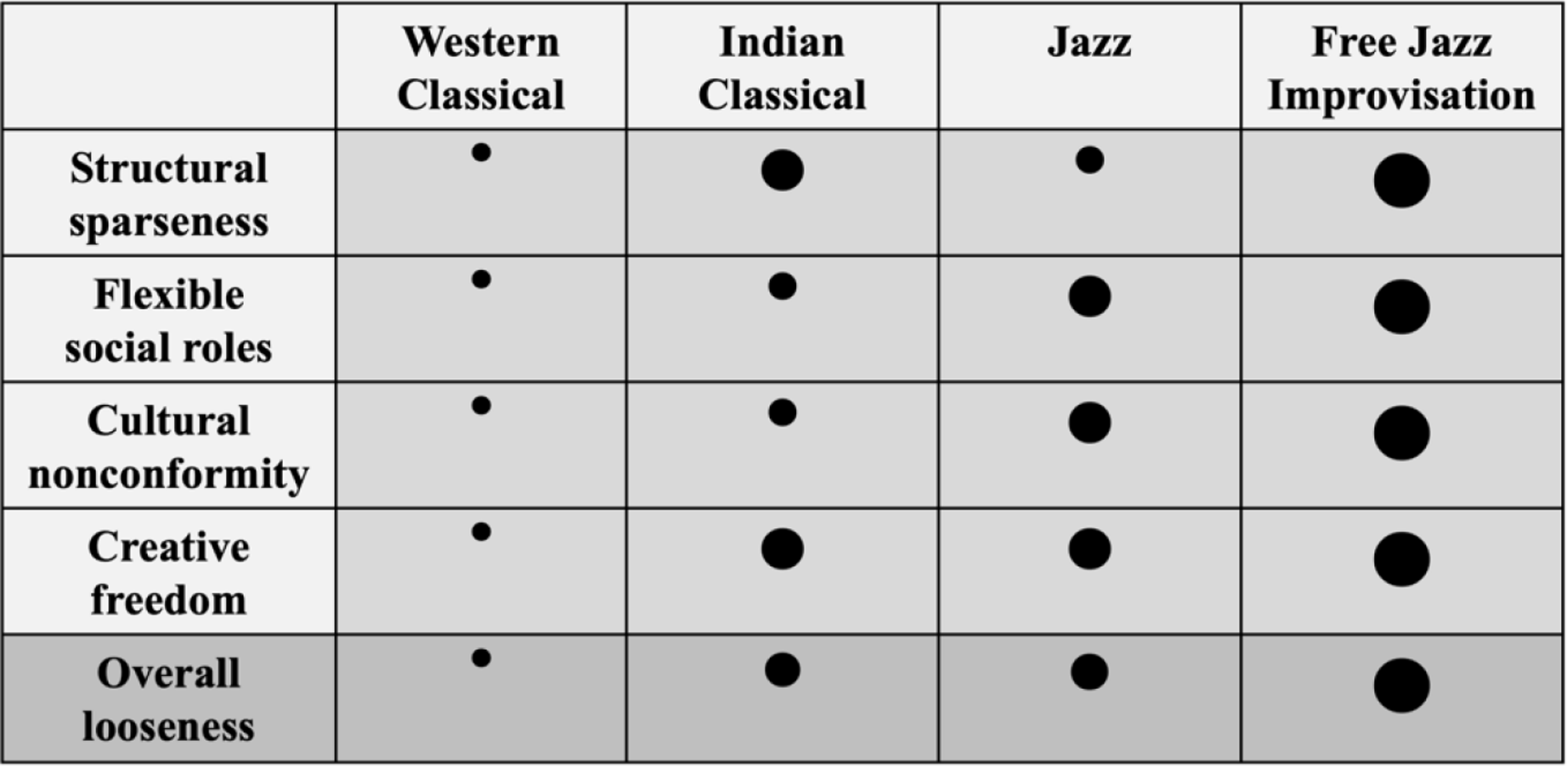

## A social lens to music

Though music is a social phenomenon, deeply embedded within a social and a cultural frame (Small, 1998; Cross et al., 2019) some historical and musicological accounts tend to treat music as a stand-alone, independent experience, not acknowledging to a sufficient extent its intricate ties with human histories and social events (Small, 1998; Taruskin, 2010). Nevertheless, many theorists examined various social aspects of music from different perspectives and foci (Small, 1998; DeNora, 2003; Finnegan, 2007; Taruskin, 2010). Small’s theorization regarding musical performance as representative of larger social dynamics, is highly pertinent to the social lens we apply in this article. Small argues that the mere concert hall venue already establishes a set of hierarchies and sub-groups by inviting a very delimited and elite part of the society and by separating the audience and players in a precisive way: seated in different parts of the hall and even entering and exiting the hall from different doorways, depending on the type of ticket purchased. Small (1998) compared between the social event of contemporary classical music (i.e., taking place in prestigious concert halls) to the completely contrasting close and intimate social event created in Rock festivals during the 1960s and 1970s. While the former gave rise to strangers sharing a physical space, yet remaining distant and alien to one another, the latter enabled new societies to evolve: groups of people who immersed in the music and with one another in a boundless and free manner. Most relevant to this piece is Small’s assertion that “those taking part in performances of different kinds are looking for different kinds of relationships, and we should not project the ideals of one kind of performance onto another” (Small, 1998, p. 49), featuring Small’s fundamental belief that different social values underlie diverse musical performances.

In a similar vein, Finnegan’s (2007) seminal work set forth the social and political structures of amateur, local music making, as reflected in a case study of the musical culture of the British city of Milton-Keynes. Through a detailed examination of the town’s musical life, Finnegan uncovers the strong social structures shaping the seemingly mundane musical events, which allow the local musicians to connect to their heritages and tradition on the one hand, and to influence the social changes and growth on the other.

Various authors among both Hindustani and Carnatic musical traditions discussed the social (Weidman, 2012) and political (Alaghband-Zadeh, 2017) representations embodied in Hindu music. Weidman (2012), for example, provided an ethnographic point of view, when reflecting on her experience of learning Carnatic music as an apprentice of her violin teacher. Such unique mode of relating, she explains, enables implicit knowledge of class, gender, and social identity to be learnt by the apprentice on sensorial and bodily levels, transcending musical mastery *per-se*. In relation to Carnatic music scholarship, Weidman accentuates the tacit knowledge of values such as social belonging, social hierarchies, and power imbalances, conveyed through the apprentice-music teacher relationship.

Several writers highlighted the unbreakable link of Jazz and free jazz improvisation to underlying social dynamics (Heble, 2000; Gazit, 2015; Liberatori, 2019). Heble (2000) presented his evolutional course of thinking of jazz playing as embedded in social structures. Firstly, Heble emphasized (mainly) black people’s ethnical and political motivations as giving rise to jazz and free jazz improvisation, as part of their social struggle for becoming emancipated from the control, hegemony and expectations of white men. However, later, the manifestations of these improvisational genres became postmodern attempts to abandon any ethical responsibility towards the audience. In that sense, Heble understands free jazz improvisation as promoting an isolation of previous social expectations. Moreover, he accentuates the ability of jazz and free jazz improvisation to evoke novel musical forms and expressions, which possibly, eventually may subscribe to alternative social forms and dynamics on their own.

Interestingly, the establishment of music therapy as a profession in the 40s and 50s of the 20^th^ century gave rise to numerous perspectives about the social and psychological aspects of music (Bruscia, 1998; Aigen, 2005). One important more recent theoretical contribution relates to the use of music in everyday life (Ansdell, 2014), and music’s inseparable connections to people’s social lives, and to the centrality of Community Music Therapy (CMT) (Stige et al., 2010). CMT focuses on the contextual and ecological meanings of music therapy, unravelling its social-cultural centrality beyond and above the music therapy room.

We will now turn to contextualizing our discussion within a musical spectrum stretching from prearranged and orally transmitted music, to highly improvisational and free genres.

## Prearranged vs. improvised music performance

In accordance with different ideas about music as deeply situated within the social matrix of life, we wish to examine the social dynamics of music performance as a consequence of the specific musical tradition it embodies, along a continuum, ranging from fully orally transmitted or notated, pre-composed and pre-arranged music to fully improvised. Although this continuum does not include all characteristics of each genre and is not central to all genres, we believe it is essential for the understanding of the social components afforded by musical interaction. Inspired by different researchers who placed different musical traditions along this continuum, [e.g., Nettl (1974) who studied improvisation or Moran (2017) who focused on the cognitive aspect of orally-transmitted compared to improvised performances], we similarly, place varied styles of performance along the orally transmitted-improvised spectrum, which assisted us in identifying key social components on which we center our discussion.

### Prearranged music

In Western Classical Music, prearranged music consists of performers communicating the semantic structures and identified features (e.g., themes, idioms, dynamics) of an existing musical piece (Thom, 2020). In this sense, according to Thom, the performer is expressing not only their individual musical intentions, but to a large extent also the composer’s original intentions, executing the piece in a recognizable manner. Thom (2020) further suggested that Western classical music entails close adherence to the performance plan, and rapid and decisive return to it in the event of an unintended deviation (e.g., a missed note or a cracked tone during singing). This reflects an underlying value of following the strict rules and orders created by the composer, which are reinforced by audience expectations (Thom, 2020). Indeed, any deviation from the ongoing musical plan will reconfirm the original aim and intention of the composer (as presented in the musical form), through the performer’s effort to seek the shortest way to get “back on track.”

Such a view of music as a formal form of art *per se* independent of any extra-musical context can be traced back to the Romantic era. This resulted in music becoming aligned as ‘artwork’ with its earlier predecessor, the plastic arts (Goehr, 1994). Goehr explained that these advances in the 18^th^ century had two main influences on the artistic evolution of music: (1) music became independent of extra-musical contents such as religion, and began to be defined and understood as pure art; (2) musicians shifted their emphasis from mere “performance” of music (which could adopt different styles and purposes) to the final product (i.e., composition) of the artistic form, the artwork, submitting to the standards of the plastic arts.

The significance of music being divided into distinct and separated categories of “artwork” (compositions) and “performance,” focusing mainly on the finalized concrete object, is highlighted by different thinkers (Moore, 1992; Bailey, 1993; Goehr, 1994; Small, 1998; Benson, 2003; Thom, 2020). Moreover, Goehr further argued that all musical genres nowadays are framed within the context of “artwork” regardless of how they were initially conceived or if their originators considered themselves as composers. For example, in Baroque music, many pieces incorporated significant improvisatory sections, which during the 19^th^ century consolidated as finalized compositions or as “artworks.” Only few musicians to date practice and perform improvisation in Western Classical pieces (e.g., Dolan et al., 2013). According to Goehr, John Cage’s famous piece 4′33, for example, was not intended by the composer as a precomposed musical work, but rather as a musical performance which bared metaphoric and conceptual significance.

### Improvisation

As opposed to prearranged music, musical improvisation consists of the instantaneous and spontaneous creation of music. Improvisation may exhibit varied degrees of musical freedom: from the expansion of existing prearranged musical elements to the generation of a new piece altogether on the spot (Nettl, 1974; Bailey, 1993; Berliner, 1994). The act of improvisation is shared by nearly all cultures and societies (Nettl, 1974; Rommen and Nettl, 2020), and underlies, for example, European vocal and instrumental ornamentation from the 16th to the 19th centuries, Indian ragas, Arab mawals and modern jazz standards.

Though manifested somewhat differently across diverse genres and cultures, improvisation seems to consistently present a certain everchanging balance between tradition and innovation, as stated by Racy (2000):

“Improvisation constitutes a merger between the familiar and the novel. It is described as an appropriate balance between satisfying musical norm and departing from it, perhaps an intermediary between compliance and defiance. In some traditions, a successful improviser must strive both to be on the innovative or exploratory edge, as well as to pre-serve the authenticity of the music.” (Racy, pp. 306–307).

Interestingly, Bailey (1993), who interviewed improvising musicians, noticed that many of them are reluctant to use the explicit term “improvisation,” and preferred “playing,” sometimes adding the specific idiom of their specialty: “playing jazz” or “playing Flamenco,” while others overtly mentioned their avoidance of the term. Bailey ties this behavior with the somewhat illusive, ephemeral, and random connotations attributed to “improvisation,” which could undermine for musicians the perceived preparedness, scholarship, training, and investment involved in such practice, according to Baily.

Nooshin and Widdess (2005) claimed that the blanket term of improvisation fails to represent the immense diversity of creative performance styles existing worldwide and embedded in various cultures. Similarly, we argue that the continuum ranging from oral and notated music to improvisation represents different practices in different cultures, and is closely attached to culture-specific social practices, needs and dynamics. To appreciate this diversity, we consider the four example musical traditions reviewed above with respect to the social contexts connected to each genre. Through this analysis we will gain a more refined appreciation of some deeper differences between diverse musical traditions and their social significance. It is important to note that some of the genres incorporate highly diverse types of improvisations and styles, however we shall focus on the central tendencies of each tradition and their respective social structures.

In order to examine the social nature of prearranged versus improvised genres, we shall now deepen our discussion by introducing and incorporating the T–L framework.

## The tight–loose paradigm in music performance

As illustrated above, the diverse genres that have evolved of musical performance reflect in many ways the political, cultural and social backgrounds and conditions, within which they emerged. However, it is challenging to compare between them due to the distinct features of each musical form and the complexity of the historical and societal context, in which they exist. In order to better align the intricate social structures underlying musical performance, we adapt the *tight-loose paradigm* used in the social sciences to the domain of music performance (Rabinowitch, 2022) as a framework for analyzing the relations between different modes of musical performance from a social perspective.

Tight–Loose theory (T–L theory, for short) was developed as a means for characterizing the essence of cultural disparities between societies (Pelto, 1968; Gelfand et al., 2011). The theory maintains that although all societies exhibit social norms, some tend to more *tightly enforce* these norms, prioritizing social order and obedience over openness and creativity. Other societies display more *looseness*, exhibiting greater openness and adaptiveness to norm violations (Gelfand et al., 2006, 2021). This single variable has proven to very effectively encapsulate important differences between distinct societies, and to serve as a valuable predictor of a variety of large-scale complex social behaviors and phenomena, as diverse as Covid-19 casualty rate (Azevedo et al., 2022; Gelfand et al., 2022) and religious beliefs (Jackson et al., 2021).

From a musical point of view, we posit that different musical traditions afford varied degrees of tightness/looseness. In particular, we identify four societal parameters, intrinsic to the music, that we suggest determine the level of tightness/looseness within each musical genre. These include *structural sparseness*, *flexible social roles, cultural nonconformity*, and *creative freedom*.

### Structural sparseness

Nettl (1974) suggested the term *structural density* to conceptualize improvisation by placing all genres, Western and non-Western, on one continuum, focusing on the density of the pre-structured elements of the improvised performance, or in Nettl’s words: “the musical models” (p. 12). While Baroque and Jazz music present a denser appearance of prearranged musical elements, Persian, Arabic and Indian music reveal a more sparse and open presence of binding musical scripts. Nettl argued that while performances incorporating thick and dense structures tend to vary less and enable limited freedom to the performers, performances which lack this kind of density provide more freedom of creativity to the players. Our theoretical analysis refines and expands Nettl’s notion of the density continuum. We argue that different modes of performance induce respective levels of social freedom that may extend beyond the musical interaction itself, affecting both listeners and performers. Applying the T–L paradigm to Nettl’s structural density concept, suggests ascribing more tightness to musical genres that closely follow pre-determined notation (i.e., Western Classical), and more looseness to musical genres that are structurally more flexible and sparse (i.e., Hindustani, and free improvisation).

While Nettl focused on the density of the pre-determined structural components alone, we wish to expand the density paradigm to include further aspects that relate to the social structures represented in the musical performance. Several studies have discussed the social nature of both notated (D'Ausilio et al., 2012; Keller et al., 2014) and improvised (Robledo et al., 2021) music-making. As a natural operationalization of human social coordination and interaction, the study of musical ensembles provides a powerful opportunity to delve into the foundations of such music-based social behavior (Keller et al., 2014; D’Ausilio et al., 2015).

### Flexible social roles

Different musical traditions endow players with varying flexibility in fulfilling their hierarchical role in the performance (e.g., soloist, accompanist, first violinist, second violinist). These roles may be fixed or interchangeable between players. When applying the T–L paradigm to this category, we attribute more tightness to musical genres that pre-determine fixed roles between the players (i.e., Western Classical and Hindustani classical music), and more looseness to musical genres that are more flexible in determining which player plays which part in a certain musical piece. Another aspect of social roles relate to the social class associated with the musical genre. As explored above, various genres are embraced or represented by different social classes in different historical eras.

### Cultural nonconformity

Cultural nonconformity can introduce different levels of strictness regarding the level of commitment players are expected to exhibit towards their musical tradition. How far does the musical genre tolerate extending and bending the musical norms while playing or improvising? What are the cultural expectations of the audience? In this category we ascribe more tightness to genres presenting high conformity and commitment to predetermined norms of performance (i.e., Western Classical and Hindustani), whereas genres that support a greater alteration of the original form and harmonic context are considered looser (i.e., jazz and free jazz improvisation).

### Creative freedom

The level of creative freedom expressed within the musical experience also varies across genres. Different musical traditions permit or prohibit varying levels of alteration in rhythm, melody, harmony, dynamics, timbre. Applying the T–L paradigm to the concept of creative freedom, we associate more looseness with genres that approve of alterations and innovations on the spot (i.e., Hindustani, jazz and free jazz). In contrast, Western Classical music exhibits more tightness in this respect.

In addition to the four aforenoted social criteria, we wish to introduce another barometer for capturing the nature of T–L among the four musical traditions: Common Group Membership (CGM). The overall depiction for each music style, displayed in the last row of Table 1, provides a relative positioning of that musical genre along the tight-loose continuum, with Western Classical music representing the tightest form of performance, and Free Jazz Improvisation being the loosest. The explicit categorization approach that we have demonstrated allows to use reasoning for performing such mapping, and to make predictions about the extent to which engagement in each musical style may affect broader aspects of social interaction. To illustrate this, we consider CGM as a feature of social interaction and conceive a hypothesis about the degree of CGM expected to be found in each form of musical performance. In other words, we wish to use our theory as a possible explanation for various in-group and out-group behaviors (Cross et al., 2019), and more specifically to utilize the level of T–L associated with each musical genre as a predictor for the quality of CGM. This analysis expands on our previous predictions regarding the effects of joint music making on CGM levels (Rabinowitch, 2022). According to Rabinowitch (2022), while tight aspects of music (e.g., the music’s rhythmic structure) can influence musicians’ higher levels of CGM (which include both positive and negative effects), it is the looser aspects of music (e.g., interpretation and improvisation) which mitigate the negative effects of CGM (e.g., blind obedience and conformity), and therefore increase the groups’ level of Tolerant Group Membership (TGM). We propose that while tighter musical genres are expected to evoke higher levels of CGM, improvisational genres will associate with lower levels of CGM and higher levels of TGM. Later in the discussion, we shall further elaborate on the levels of CGM/TGM afforded by the various musical traditions and genres.

Next, we shall depict the *musical* T–L characteristics (i.e., the societal components) of the four musical genres discussed so far (Western Classical; Hindu music; Jazz; Free Jazz Improvisation). In addition, Table 1 provides a simplified illustration of the quality of each societal component as well as the overall nature of the four societal components (i.e., how tightly or loosely they manifest within each musical genre).

## Mapping different musical traditions to tight-loose social tendencies

As an example for how to characterize different musical traditions and compare between them in terms of tight-loose social tendencies we describe a preliminary mapping of different musical styles according to the social parameters we have introduced, positioning them on to the T–L continuum. We portray each of the musical traditions from low to high along the 4 social categories, *structural sparseness*, flexible *social roles, cultural nonconformity*, and *creative freedom* (Table 1). This framing is based on our overall familiarity with the different musical styles and on informal interviews that we have conducted with professional performers from each genre. It thus provides a general qualitative T–L charting of the music styles that could later on be fine-tuned, for example, through questionnaires and expert judgements.

When focusing on the *structural sparseness* of each musical structure, one can notice there is no clear gradient from tight to loose. *Western Classical* performance receives the lowest characterization on structural sparseness due to its closed, notated, and highly restricted form. *Hindustani music*, exhibits more spacious musical structures than *Jazz* music. This is due to the tighter adherence to jazz standards, even during improvisation. Although Hindustani music abides to extremely strict guidelines (e.g., a particular Raga or Makam), most of the musical structures are created on the spot, while referencing the pre-determined musical materials as foundational elements, rather than a form. Free jazz improvisation receives the highest account on structural sparseness, as this genre does not exhibit any pre-determined structural restrictions.

*Flexible social roles* displays a clear gradient (Table 1), growing from the strictest social roles, manifested in *Western Classical* performance, to the loosest within *Free Jazz Improvisation*. While in *Western Classical music* the roles of musicians are entirely predetermined by their seat in the orchestra or role in a chamber music ensemble,^2^ a free jazz improvisation performance does not impose any social restrictions on the players and allows them to continuously negotiate the leadership over the performance. This was nicely demonstrated by Hadar and Amir (2021) who studied joint improvisations between jazz and free improvisors. One of their key findings relates to the wide range of relationships enabled between performers of this genre. Hadar and Amir (2021) specified seven different types of relationships present among jazz and free improvisors, emphasizing the players’ tendency to rapidly switch between close intimately, to “fighting” and “teasing” each other on stage.

From similar considerations, we considered *Jazz* performance also high on flexibility of social roles. The lenient roles experienced in jazz performance was highlighted by additional researchers (Berliner, 1994; Monson, 1996; Aigen, 2013) and seems to play a major role in this musical genre. *Classical Indian* performances do not follow pre-specified partitures, thus providing a wider basis for musical choice, also on the social hierarchy dimension. However, following strong traditions and performance conventions, this genre shows a tendency to assign players with the traditional roles ascribed to their instruments, and even when these constraints are relaxed, a clear plan for switching roles is predetermined, and is never spontaneous.^3^ Another point to consider relates to the social class, in which the respective musical genre had evolved. For example, while nowadays both Western classical music as well as jazz and free jazz improvisation are associated with elite and bourgeoise parts of society (Small, 1998), the genres originated in quite differing political ecologies. Both Western Classical music and Hindu music originated in religious practices, and to date subscribe to the original musical forms to a certain extent. In contrast, Jazz and Free Jazz Improvisation originated in secular and rebelling societies.

On a different note, one should consider the influence of the players’ musical proficiency on the social interaction enabled between them. While this component is relevant to all musical genres to a certain extent, clearly, the more restrictions and cultural conformity the players are subjected to, the greater skill level they have to develop. This subscribes both to the social layers associated with classical genres as well as to the possible competition and feeling of hierarchy and estrangement between the players, as pointed out by Small (1998). Nevertheless, studies about jazz players (Berliner, 1994; Hadar and Amir, 2021) revealed the great emphasis jazz players place on their musical partners’ professional level, and their noncompromising on less than top notch musical partners to improvise with. In this sense, it seems that players’ skill level is an important factor in all traditions and should be taken into account. In essence, one may think of skills as a catalyst of T–L alignment. We believe that the level of skill presented by musicians is associated with the breadth and depth of their T–L spectrum. Therefore, we postulate that musicians with greater skill will present a broader T–L continuum for the various musical genres they practice.

As for *cultural nonconformity*, perhaps the most striking feature of this dimension is its disparity between being either highly strict and conformist or highly loose and nonconformist (Table 1) among the four traditions. *Western Classical* performance is strongly driven by tradition through clear unequivocal notated instructions, thus appeared as encouraging high conformity regarding cultural expectations. *Hindustani and Carnatic classical traditions* are similarly guided by cultural expectations, perhaps even more explicitly and specifically. This complies with Pudaruth’s (2016) description of the complex systems Hindustani players subscribe to when performing their orally transmitted music. Therefore, though differing in many respects, we deem Western Classical and Hindustani music to share a similar level of strong sentiment over their long living traditions, which appropriately accounts for their label as “classical.” In contrast, *Jazz* and *Free Jazz Improvisation* can be placed on the other extreme of the cultural nonconformity range, as one of the main characters of jazz performance lies within the players’ ability to stretch the boundaries of any norm or tradition, or to completely defy them, as in the case of *Free Jazz Improvisation* (Berliner, 1994; Lewis, 2002; Niknafs, 2013; Schroeder, 2019). We wish to add one caveat, relating to the cultural expectation of Jazz audience regarding the performance’s structure (i.e., including solo versus ensemble part). In this sense, although jazz playing grants musicians many degrees of freedom in several aspects of the piece performed (e.g., the melody, harmony, timbre, arrangement), most players conform with the cultural expectation of the structure of the performance, by alternating between solo and ensemble parts, leaving it almost exclusively untouched throughout this genre’s lifetime.

When considering the level of *creative freedom* across all four musical genres, we note a sharp contrast between the *Western Classical* genre to all the rest. While Western Classical performance affords the least creative freedom to players, who are autonomous in only subtle musical elements such as dynamics and tempo (Thom, 2020), all other genres seem to incorporate high levels of creative freedom. We posit that *Hindustani music* and *jazz music*, all share a similar level of creativity enabled within their performance. In all three genres the players are expected to provide a constant flow of new musical materials, as the quality of the performance relies greatly on their ability to create novel music in the moment.

While players in *Hindustani* music are in fact composing their piece “in the moment,” they are bound to highly strict guidelines which eventually narrow their freedom of musical choice, e.g., the fixed patterns of the Hindustani Tala (Pudaruth, 2016). *Jazz* players, on the other hand experience a greater range of independent exploration while improvising but are yet bound to a musical form (i.e., the jazz standard) that serves as a basis for their improvisation. Ultimately, *Free jazz improvisors* are expected to use their creative resources in full and are expected to create in the moment the form and musical substance in the most free, surprising and novel way they can imagine (Berliner, 1994; Lewis, 2002; Niknafs, 2013; Schroeder, 2019).

## Common group membership and musical interaction

Common group membership (CGM) relates to a person’s sense of belonging, affiliation, rapport, and obedience to a certain social group, and is known to effect positive social behavior towards group members (in-group) and negative behavior towards non-members (out-group) (e.g., Tajfel, 1970; Branscombe and Wann, 1992). It has been suggested that group music performance entails CGM through, for example, interpersonal synchrony (Tunçgenç and Cohen, 2016; Cross et al., 2019; Bente and Novotny, 2020), the continuous temporal alignment of players according to the rhythmic structure of the musical piece, and their collective focus on a shared beat (e.g., Savage et al., 2021). While CGM may be considered as a positive driving force for prosocial behavior, it may also comprise certain negative sides, such as increased conformism or exclusion of non-group members (Rabinowitch, 2022). We argue that CGM is not fixed and can vary according to the social dynamics present within the interaction. Indeed, a looser attitude could produce a more tolerant form of CGM (TGM), which we term tolerant group membership, entailing a weaker sense of inclusion on the one hand, and high freedom and creativity on the other (Rabinowitch, 2022). Conversely, tighter social interactions may lead to more stringent CGM, entailing for example, strong social bonding and inclusion, but lower creativity and individuality. We thus conjecture that different musical styles could associate with different degrees of CGM. In particular, our analysis predicts that playing Western Classical music, for example, should give rise to stricter CGM among the players, whereas Free Jazz Improvisation should drive CGM towards the more lenient TGM. Other musical styles should have an intermediate effect. Therefore, the impact of musical performance on social interaction is music-style dependent.

## Conclusion

Music is a prevalent human activity, extending from deep social roots. We have sketched how different styles and genres of music performance may have emerged from distinct social climates and practices, and have proposed a systematic method for mapping different types of music according to four major dimensions of classification, *structural sparseness*, *flexible social roles, cultural nonconformity*, and *creative freedom*. To this end we have adopted the notion of tight vs. loose adherence to social norms and practices widely used in the social sciences, and characterized each musical form in terms of tightness/looseness, deriving an overall score for each music type along this continuum. This approach enables to compare and distinguish between different musical traditions and styles and their impact on social dynamics, discerning between musical styles that are predicted to elicit tighter interactions (e.g., stricter group membership), such as Western Classical music, and those promoting looser interactions (e.g., tolerant group membership), such as Free Jazz Improvisation. Therefore, music performance occupies a broad spectrum of styles that originate from and evoke a range of fundamental social dynamics.

Further research is required in order to understand how such advantages might be deployed in varied contexts such as music, educational and therapeutic settings. For example, what would be the ideal type of music to encourage creative and independent learning and what musical style would support group work and cooperation? Building on the theoretical notions of this paper, we would hypothesize that whereas styles that are considered as looser on the musical T–L continuum may promote student creativity, other styles that are assumed tighter on the continuum, calling for stricter adherence to musical roles and arrangements, may encourage social bonding and group learning but not creativity or individuality. Several studies imply a connection between playing Western Classical music and a discrete type of social tendencies. For example, Müller et al. (2018) pointed to the prosocial effect and increased levels of in-group cohesion associated with singing in choirs (which mostly perform Western Classical music). Focusing on Western traditions, emphasized the tension between several social and organizational components underlying small musical ensembles, namely stability and change, collectivity versus individuality, and maturity versus emergence (Pennill and Dermot, 2021). Our analysis provides an underlying explanation for the social matrix embedded in such musical contexts, suggesting that playing notated and oral music embodies tighter social structures, which in turn might influence the way music groups behave and develop together.

Interestingly, as specified by Small (1998), while originating in a different ecology, nowadays, (tight) Western classical music exists mainly among privileged, liberal, and democratic societies (i.e., societies which are characterized as loose according to Gelfand et al., 2021). Looser-classified musical genres on the other hand, such as classical Indian music, are highly prevalent among more traditional and tight classified societies. Several explanations might account for this contradiction, one of them, perhaps pertaining to the evolution of music’s role in society (Goehr, 1994; Davies, 2001). For example, while classical music seemed to represent the Catholic laws and traditions for many years (Taruskin, 2010), it might have become a privileged and almost individual artistic choice in the current boundless capitalistic musical market (Small, 1998). In other words, the music score remained the same but the musical performance (i.e., Small’s concept of *musicking*) might represent different values and uses along its life span.

Conversely, group (Ruud, 1998; Ansdell, 2014) and dyadic (Hadar and Amir, 2021) improvisation has been described by several researchers as a liminal experience, enabling players to establish close relationships, and requiring their acknowledgment of each other’s individual identities, in a way that resonate Buber’s concept of I-Thou (Buber, 2000). Our study argues that the benefits derived from participating in group improvisation might subscribe to the level of looseness enabled between players, which lays the basis for players’ unique and inner voices to unfold. Such processes are also highly pertinent to musical work as described in the music therapy context (Bruscia, 1998; Nordoff and Robbins, 2007; Hadar and Amir, 2021), whereby musical improvisation is utilized to create a unique social bond between client and therapist and to enable clients’ individual voices to be revealed. Different conditions and methods might be at play to enable such therapeutic processes, e.g., creating a safe and predictable environment for clients (Bruscia, 1998), engaging in deep listening to clients’ sounds, movements, and general expressions (Nordoff and Robbins, 2007; Hadar and Amir, 2021). We argue that a fundamental ingredient for therapeutic progress may be the level of looseness enabled between client and therapist. Moreover, we feel that further research should focus on the dialectical movement between tight and loose components in music therapy, and its significance to therapeutic outcomes in music therapy.

Ultimately, this paper aimed to dissect various social structures embedded within musical performance as reliant on the extent of tightness or looseness of specific social dimensions. We believe that further research could allow greater understanding of the possible social, emotional, cultural, and so forth effects afforded by different performance styles. While one type of performance (i.e., loose) might advance freedom of individual exploration and self-expression, participating in a different style (i.e., tight) might promote feelings of belonging and embracement within a larger group or tradition.

## Data availability statement

The original contributions presented in the study are included in the article/supplementary material, further inquiries can be directed to the corresponding author.

## Author contributions

All authors listed have made a substantial, direct, and intellectual contribution to the work and approved it for publication.

## Funding

This research was supported by the Israel Science Foundation (No. 100008920) awarded to T-CR.

## Conflict of interest

The authors declare that the research was conducted in the absence of any commercial or financial relationships that could be construed as a potential conflict of interest.

## Publisher’s note

All claims expressed in this article are solely those of the authors and do not necessarily represent those of their affiliated organizations, or those of the publisher, the editors and the reviewers. Any product that may be evaluated in this article, or claim that may be made by its manufacturer, is not guaranteed or endorsed by the publisher.

## Footnotes

^1^For the purpose of the current research, we treated "Free jazz improvisation" in its ultimate manifestation: as freed from any musical idiom or convention. We wish to acknowledge that along its evolution, this genre grew to incorporate various musical expectations, both from the audience’s perspective, as well as from the players themselves (Pras, 2015). However, as in the rest of this study, we focused on the salient tendencies of this genre, with the intent to capture the underlying social structures associated with it.

^2^In effect, Western classical music presents an abundance of social conditions, and presents a diversity of situations afforded by different types of musical performances (e.g., playing in an orchestra, singing in a choir, or playing in a chamber music group). Nevertheless, we wish to stress that an underlying principle is shared by all Western classical performances today, and that is, they all establish pre-determined roles, and adherence to the (many, changing and complex) social structures determined through the musical arrangement.

^3^This was specifically highlighted in personal correspondence with with Anagha Bhat regarding Hindustani music.
